# Effects of singing bowl exposure on Karolinska sleepiness scale and pupillographic sleepiness test: A randomised crossover study

**DOI:** 10.1371/journal.pone.0233982

**Published:** 2020-06-01

**Authors:** Melanie Bergmann, Stefan Riedinger, Ambra Stefani, Thomas Mitterling, Evi Holzknecht, Peter Grassmayr, Birgit Högl

**Affiliations:** 1 Department of Neurology, Medical University of Innsbruck, Innsbruck, Austria; 2 Department of Therapeutic Radiology and Oncology, Medical University of Innsbruck, Innsbruck, Austria; 3 Department of Neurology 1, Kepler University Hospital, Linz, Austria; 4 Bell Foundry Grassmayr, Innsbruck, Innsbruck, Austria; University of Rome Tor Vergata, ITALY

## Abstract

**Background:**

The aim of this study was to investigate the effects on subjective and objective sleepiness of a stay above a large struck singing bowl compared to a relaxation period in a silent singing bowl.

**Methods:**

Fifty-eight healthy subjects were recruited for the study, 48 participated on two days, one week apart, during the same timeslot. The Karolinska sleepiness scale was used to evaluate current subjective sleepiness, and the relative pupillary unrest index to assess objective sleepiness. In this randomized cross-over study, the intervention consisted of a 20-minute stay in a hammock while the singing bowl, positioned beneath, was struck seven times. The controlled comparator was a 20-minute stay in the same hammock above the singing bowl, but without being struck. After these two interventions subjective and objective sleepiness were re-evaluated.

**Results:**

The mean relative pupillary unrest index values after relaxation in the struck and silent singing bowl groups were 0.74 and respectively 0.71 (p = 0.460). The median Karolinska sleepiness scale value after relaxation with the struck singing bowl was 3 compared with 4 (p = 0.041) for the silent singing bowl.

**Discussion:**

This study evaluated the influence of a struck singing bowl on sleepiness during daytime. Subjective sleepiness was significantly lower after relaxation above a struck singing bowl. After gender stratification, the difference was still significant in women. Objective sleepiness was not different in both groups. Finally, we can only speculate if women may be more susceptible to subjective improvements in case of sleepiness and show another perception of relaxation in a struck singing bowl compared to men.

## 1. Introduction

Ancient instruments such as Tibetan (also called Himalayan) singing bowls were used for religious and spiritual ceremonies, including shamanic journeying and meditation [1]. Tibetan singing bowls are made of metal alloys and used by Tibetan monks for spiritual ceremonies [2]. Singing bowls produce harmonic sounds as well as vibrations that can be felt when in close proximity. Anecdotal evidence supports the value of singing bowls in relaxation and meditation [3, 4]. One observational study has found that Tibetan singing bowl meditation is useful in decreasing tension in individuals who have not previously practiced this form of meditation [5]. A randomized crossover study has investigated the effects of relaxation with a singing bowl in 51 patients. They found a greater decrease in systolic blood pressure and heart rate after relaxing with the singing bowl compared to relaxing in silence [6]. In this study, we aimed to investigate the effect of a 20-minute relaxation period with an activated singing bowl on subjective and objective measures of sleepiness, compared to the effect of a 20 minutes relaxation with a silent singing bowl. To assess the subjective sleepiness we decided to use the Karolinska Sleepiness Scale (KSS) and for the objective sleepiness the Pupillographic Sleepiness Test (PST).

## 2. Methods

This study had a randomised crossover design. We prospectively included 58 adult participants, between November 2014 and March 2015, who underwent two assessments on two consecutive weekends. The participants were recruited among acquaintances of the authors. Participants were informed about the aim of this study, but not regarding predicted outcomes.

Due to the size of the singing bowl, the assessments took place at the bell foundry Grassmayr in Innsbruck, Austria. The investigations took place at weekends to guarantee maximum privacy and a quiet environment for the participants.

Data including age, weight, height, mean reported sleep duration on work- days, Epworth Sleepiness Score (ESS), prior medical history, intake of alcohol, caffeine, nicotine or medication, were collected via semi- structured interview. Participants aged between 20 and 60 years old were eligible for the study. Exclusion criteria included a body mass index > 30 kg/m^2^; report of less than six hours of sleep the night before the assessment; insomnia (defined as subjective difficulty to initiate and/ or maintain sleep); excessive daytime sleepiness (ESS > 10/24 points) [7]; known pupillary afferent or efferent lesions; the consumption of more than three glasses of alcohol the prior evening; and the intake of caffeine, nicotine, benzodiazepines, or stimulants the morning of the assessment.

The KSS and the PST were used to measure the participants’ current sleepiness. The KSS is an EEG-validated scale for evaluating state sleepiness [8, 9]. The PST is an objective method for rating sleepiness via detection of pupillary oscillations [10], which are measured and quantified through automated analysis. Sampling frequency is 25Hz with a spatial resolution of 0.05mm (PST, by AMTech Dossenheim, Germany, F^2^D-Fit-for-Duty). PST has been validated as a method for evaluating sleepiness [11–15]. Results of the PST are based on the pupillary unrest index (PUI). Normative PUI values have been published for adults between the ages of 20 and 60 years old [15] as well as for schoolchildren [16].

In general, studies have supported the PST as a reliable tool for determining daytime sleepiness and alertness levels. Enhanced pupil unrest indices have been found in healthy sleep-deprived subjects [12, 14], as well as in patients with hypersomnia suffering from narcolepsy [13, 17] or obstructive sleep apnoea syndrome [13, 18, 19].

In this study, the device, used to register pupillary activity, was the PST, F^2^D Fit-for-Duty (AMTech, Pupilknowlogy GmbH, Dossenheim, Germany). The outcome parameter was the rPUI. Current validation studies of the rPUI have not been published so far.

Approval was obtained from the ethical committee at the Medical University of Innsbruck, Austria. All participants granted written informed consent in accordance with the Declaration of Helsinki.

### 2.1 Study design

Participants underwent two investigations on two consecutive weekends, conducted at the same time interval (between 11:00 and 18:00) in order to minimize circadian effects. The duration of each investigation was about one hour. By means of a coin toss, participants were randomized into one of the two groups regarding the order of the investigations.

#### 2.1.1 First experimental session

For the first group of participants, the singing bowl was used in the first investigation whereby participants lay down in a hammock over the singing bowl. The singing bowl was struck seven times (entire duration 210 seconds), with intervals of 30 seconds, using an automatic rope winch. After the striking of the bowl, the volunteers spent 20 further minutes lying over the bowl relaxing. Subjective (KSS) and objective (rPUI) sleepiness parameters were measured before and after the relaxation in the struck singing bowl.

#### 2.1.2 Second experimental session

Participants in the second group were given an opportunity to rest without the sound waves of the giant singing bowl. They spent 20 minutes in the hammock over the singing bowl but the bowl was not struck. Subjective (KSS) and objective (rPUI) sleepiness parameters were measured before and after the relaxation in the silent singing bowl; the measurements were then compared.

**Fig 1:**
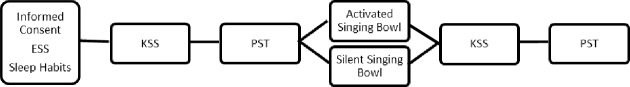
Course of the study procedures.

The course of the study procedures is listed in Fig 1 (Fig 1) and in the study protocol (http://dx.doi.org/10.17504/protocols.io.bge9jth6).

Participants changed groups when they returned at the following time point. Both, during the periods in the singing bowl (Peltor ^TM^ Optime ^TM^ II, 3M, St. Paul, Minnesota, USA) and the pupillography (Peltor ^TM^ Optime ^TM^ I, 3M, St. Paul, Minnesota, USA), participants were provided with earmuffs to reduce ambient noise level.

### 2.2 Materials

#### 2.2.1 Singing bowl

In 2012, the bell foundry Grassmayr, a traditional company in Innsbruck, Austria, cast a singing bowl made of bronze (diameter 176 cm, height 65 cm, weight 1200 kg). An expert review, conducted at the company’s behest, confirmed the harmlessness of the noise generated by the singing bowl [20].

**Fig 2:**
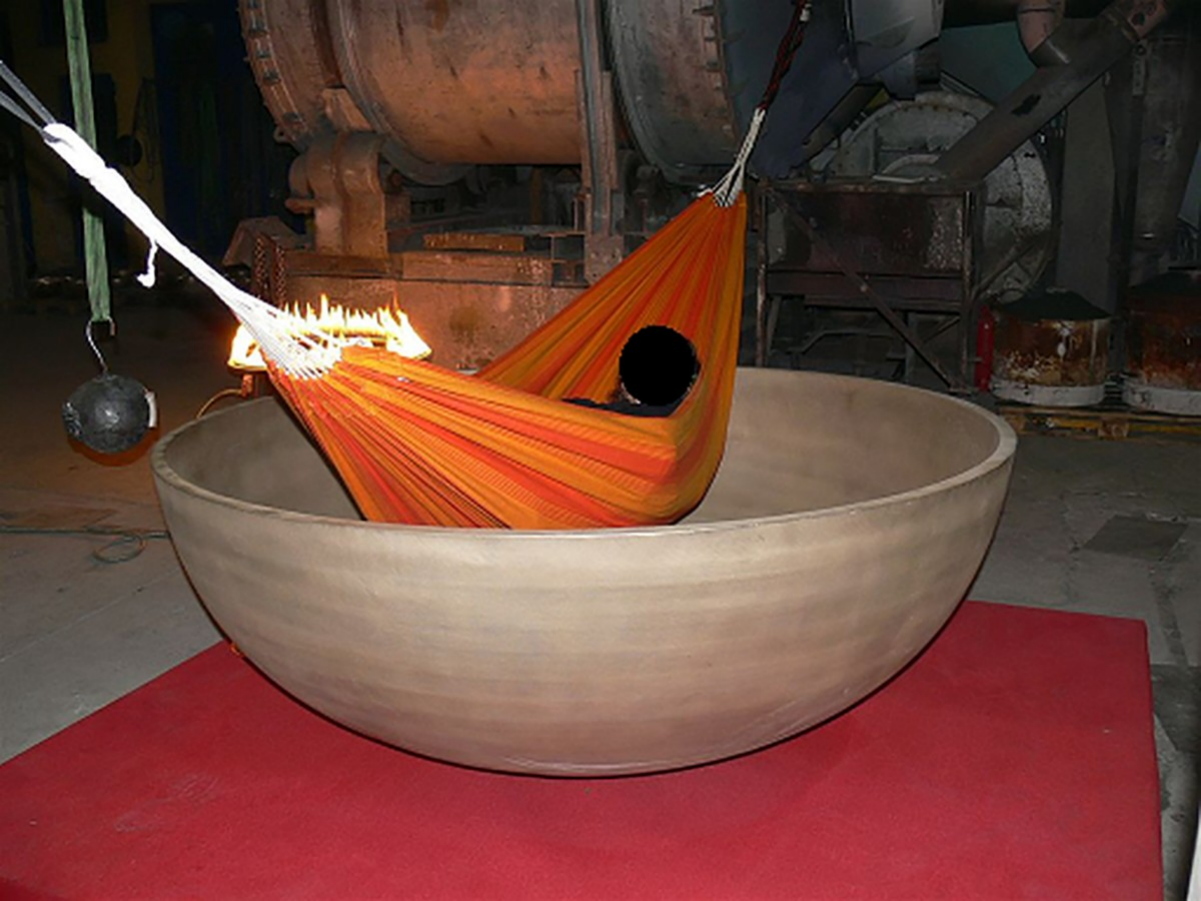
Singing bowl with a hammock and a rope winch (on the left-hand side of the picture).

**Fig 3:**
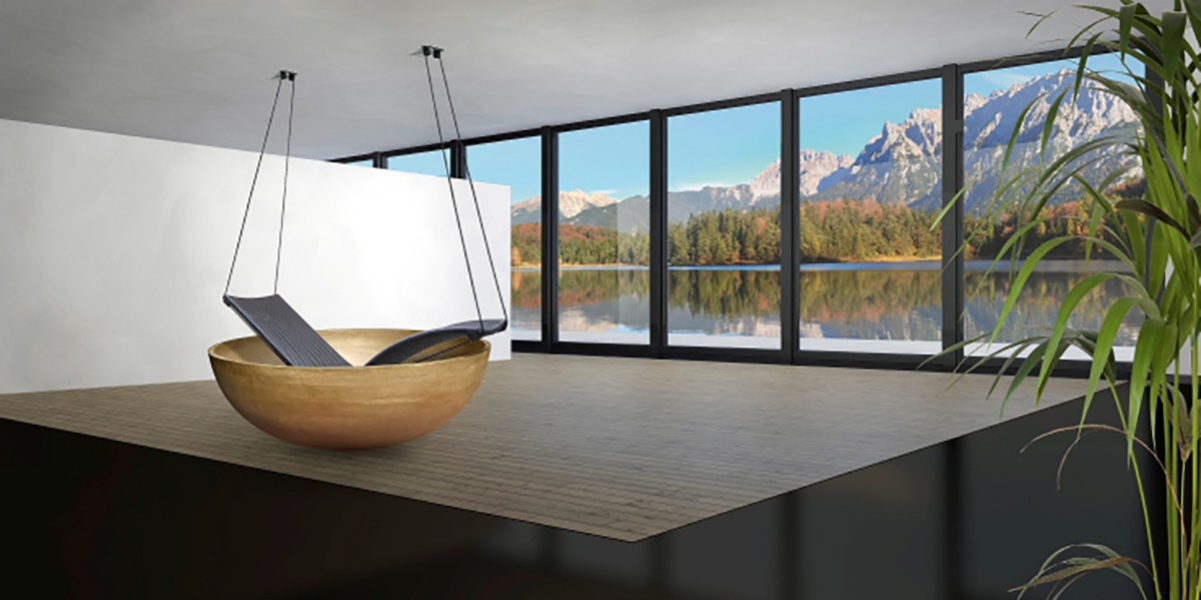
Singing bowl with a hammock.

The singing bowl was prepared on the factory work floor on a wooden platform (Figs 2 and 3).

#### 2.2.2 Karolinska sleepiness scale (KSS)

The KSS [7] measures the subjective level of sleepiness at a particular time during the day. It measures situational sleepiness via self-reported measurement (1 = extremely alert, 3 = alert, 5 = neither alert nor sleepy, 7 = sleepy, but no difficulty remaining awake, 9 = extremely sleepy or fighting sleep).

#### 2.2.3 Pupillographic sleepiness test (PST)

Wilhelm and colleagues developed the pupillographic sleepiness test (PST), which uses infrared video pupillography to record spontaneous oscillations of the pupil size in the dark over a 11-minute period [9]. Spontaneous, slow oscillations of the pupil diameter (‘fatigue waves’) in complete darkness were first observed by Lowenstein et al. in 1963 and indicate a reduced level of wakefulness [21]. The autonomic nervous system regulates pupillary size. Central modulation of sympathetic and parasympathetic activity results in dynamic equilibrium of the pupillary size. Increases in sympathetic activity usually accompany central inhibition of parasympathetic activity. A noradrenergic pathway mediates central inhibition and connects the locus coeruleus to the Edinger Westphal nucleus. The second pathway connects the A1/A5 nuclei of the brainstem to the Edinger Westphal region and is GABAergic [13, 22].

Registration of the pupillary activity was carried out using a portable measuring device (PST, F^2^D Fit-for-Duty, AMTech, Pupilknowlogy GmbH, Dossenheim, Germany).

The testing room was completely dark. Participant and investigator sat facing each other. To minimize interference from mental or physical activity, subjects rested for at least 15 minutes before the PST recording. All participants were asked to look at a small infrared light source (wavelength 880nm) for the duration of the investigation. Goggles with infrared filters were worn to exclude light influences that could provoke light-induced oscillations potentially mimicking sleepiness effects.

Patients were briefed with the following instructions: “This measurement is going to last 11 minutes. During the measurement, it will be dark and quiet in the room. We will not talk to you until the recording is completed. Please look in the direction of the red light. You do not need to focus on it sharply. Please do not perform mental arithmetic or try to solve problems in your mind. Just relax and look straight ahead. We will set up the measurement now and tell you when it starts.” [14, 23].

The calculation of the pupil diameter was developed by the pupil research group at the University Eye Hospital Tübingen and patented by the University of Tübingen (DPA 5402P137). Recording of the oscillations of the pupil diameter was performed every 40 ms (sampling rate: 25Hz) with a spatial resolution of 0.05mm.

The whole test duration of 11 minutes was divided into eight time segments, each lasting 82s, consisting of 2048 data points per PST measurement. The PUI (mm/min) as the main outcome parameter is the sum of absolute changes in the pupil diameter (in mm) based on a sampling frequency of 1.5625Hz. Higher values indicate increased daytime sleepiness. Further information and details are available in Lüdtke et al. [9, 12].

Smaller pupils may have a smaller possible constriction range than larger pupils. Therefore, the rPUI was introduced and includes the baseline pupil size. The rPUI was defined as the PUI divided by the baseline pupil diameter [12].

### 2.3. Statistical analysis

Statistical analyses were performed with the Statistical Package IBM SPSS Statistics Version 24 (SPSS Inc., Chicago, Illinois, USA). Normal distribution was assessed with the Kolmogorov-Smirnov-Normality Test. Patient characteristics are presented as median, range and interquartile range (IQR), when not normally distributed, as means and standard deviation (SD) when normally distributed. Group comparison was carried out with the Mann-Whitney-U-Test for parameters which were not normally distributed, and with the T-Test for unpaired samples in case of normal distribution.

For the group- gender- adjusted comparison between the relaxation periods in the struck and silent singing bowl, the repeated measurement ANOVA with the covariate gender was applied of rPUI, Friedman test was used for the KSS. For the comparison of subjective sleepiness (KKS), before and after the relaxation in the activated and silent singing bowl, we additionally performed the Wilcoxon signed rank test for related samples. A p-value <0.05 was considered statistically significant.

## 3. Results

### 3.1. Demographic data

Of the 58 participants initially recruited, 10 had to be excluded: for three participants the technical quality of the PST recording was poor, four refused to undergo the second examination, one violated the caffeine limitation, one had a body mass index > 30 kg/m^2^, and one was sleep-deprived.

The remaining 48 healthy volunteers (25 men (52.1%), 23 women (47.9%)) had a median age of 31 years (range 20–59) and a mean body mass index of 22.8 ± 2.5 kg/m^2^. The mean Epworth Sleepiness Score was 6.3 ± 3.1. The reported mean sleep duration on work- days amounted to 8 h 50 minutes ± 1.2. Further details are listed in Table 1.

**Table 1:** Demographic and sleep parameters of the study participants.

N (%)	Study Population, 48 (100)	Men, 25 (52.1)	Women, 23 (47.9)	p-value
Age, years, median (range)	31.3 (20–59)	33.5 (20–59)	29.8 (20–59)	0.772
Body Mass Index, kg/m^2^, mean (SD^a^)	22.8 ± 2.5	23.2 ± 2.7	22.4 ± 2.4	0.257
Epworth Sleepiness Score, mean (SD^a^)	6.3 ± 3.1	5.6 ± 3.2	7.1 ± 2.9	0.098
Reported Sleep Duration on work- days, h, mean (SD^a^)	8.8 ±1.2	8.9 ± 1.3	8.8 ± 1.1	0.837

SD, standard deviation

### 3.2. Karolinska sleepiness scale and relative pupil unrest index (rPUI)

Comparison between KSS before and after the application of the singing bowl revealed no differences in the whole sample (p = 0.709) and in the male participants (p = 0.117), but significant differences among females (p = 0.029). There were significant differences in KSS values after the relaxation period in an activated singing bowl in the whole study population, as well as between women and men. rPUI did not differ significantly in either group. A comparison of subjective and objective sleepiness measures are shown in Table 2.

**Table 2:** Subjective and objective sleepiness.

n = 48	Relaxation in the activated singing bowl	Relaxation in the silent singing bowl	Men n = 25	Relaxation in the activated singing bowl	Relaxation in the silent singing bowl	Women n = 23	Relaxation in the activated singing bowl	Relaxation in the silent singing bowl	p-value* treatment	p-value gender	p-value treatment * gender	p-value total	p-value women	p-value men
**KSS**^a^, median (range) IQR^b^			**KSS**^a^, median (range) IQR^b^			**KSS**^a^, median (range) IQR^b^								
before	3 (1–7) IQR 3–4.75	3 (1–7) IQR 3–5	before	3 (1–6) IQR 2–4	3 (2–6) IQR 3–4	before	3 (2–7) IQR 3–5	4 (1–7) IQR 3–6				0.229	0.359	0.607
after	3 (1–7) IQR 2.25–5	4 (1–7) IQR 3–6	after	4 (1–7) IQR 3–6	4 (2–7) IQR 3–6)	after	3 (1–7) IQR 2–4	4 (1–7) IQR 3–6				0.041	0.031	0.629
delta	0 (-5-3) IQR -1-1	0 (-6-3) IQR -1-1	delta	0 (-5-3) IQR -1.5–0.5	0 (-4-3) IQR -2-0	delta	1 (-5-3) IQR 0–2	0 (-6-3) IQR -1-1				0.324	0.167	1
**rPUI**^c^, mean (SD^d^)			**rPUI**^c^, mean (SD^d^)			**rPUI**^c^, mean (SD^d^)								
before	0.74 ± 0.37	0.71 ± 0.35	before	0.76 ± 0.44	0.69 ± 0.33	before	0.73 ± 0.29	0.73 ± 0.37	0.399	0.999	0.386			
after	0.74 ± 0.32	0.71 ± 0.30	after	0.75 ± 0.34	0.71 ± 0.30	after	0.71 ± 0.30	0.70 ± 0.29	0.460	0.773	0.654			
delta	0.01 ± 0.21	-0.35 ± 0.28	delta	0.01 ± 0.23	-0.09 ± 0.35	delta	0.01 ± 0.20	0.02 ± 0.18	0.416	0.240	0.296			

^a^KSS, Karolinksa Sleepiness Scale

^b^IQR, interquartile range

^c^rPUI, relative pupil unrest index

^d^SD, standard deviation

*P-values are adjusted for gender.

## 4. Discussion

We evaluated the effect on subjective and objective sleepiness of a 20-minute relaxation period over a large singing bowl that was struck, compared to the effect of a similar period of relaxation in the same position over the bowl, but in silence. While measures of subjective sleepiness differed between the two investigations, no difference was found for objective measures.

There was a significant difference in the subjective sleepiness (KSS) in the whole study population and in the subgroup of women between the relaxation periods with the activated and the silent singing bowl. Subjective sleepiness was lower after relaxation with the struck singing bowl. In the subgroup of men, we found no difference between the groups. Objective sleepiness measured with the rPUI did not differ between the groups. Elsewhere, the PST has been shown to be an appropriate tool for measuring sleepiness. Such discrepancies between subjective and objective sleepiness measurements have already been reported in the published literature [24–26].

When a separate analysis for gender was carried out, it became apparent that this effect seemed to be driven only by women.

The separate analysis for gender was carried out, because it has been previously reported in the literature that women use more often complementary and alternative medicine (CAM), including acupuncture, homeopathy, traditional healers, chelation, herbal supplements, chiropractic/ osteopathic body therapies, massage, biofeedback, meditation, yoga, Tai Chi/ Qi Gong and energy therapies, compared to men. [27] Two large surveys with 4,645 and 7,919 adults, suffering from migraine and severe headache, and from arthritis, respectively, reported a more frequent usage of CAM in women than in men. [27, 28]

Furthermore, a study of the general US population reported that the higher utilisation rates of CAM among females is mainly due to their positive perceptions about CAM and its impact on health and wellbeing. [29] Of note, it has been reported that cognitive behavioural therapy and metacognitive training (combining psychoeducational components and cognitive behavioural therapies) seems to be more effective in women. [30, 31]

We can only speculate if the women in our group were more perceptive to subjective improvements, or were more susceptible to delivering supposedly expected or positive results. A large interview- based survey found that women who used CAM were more likely to report positive outcomes and greater benefit compared to male CAM users. The authors suggested that one possible explanation for this finding may be that women are more responsive to the effects of CAM. [29] Gender differences in the perception of different symptoms have been reported in the case of pain perception. Women reported more intense and frequent pain and were more likely to experience pain in multiple body regions. [32, 33] Furthermore, it has been reported that women benefit more from multimodal pain therapy, including cognitive behavioural therapy, than men. [34]

Alternatively, in our study the subjective effect might not be evident in men because of the smaller sample size of this subgroup.

To summarize, we found a positive effect of the struck singing bowl on subjective, but not objective sleepiness measurements in the whole study population, mainly driven by women. Regarding the evaluation of subjective sleepiness, a limitation is, that complete blinding was not possible, because volunteers did perceived the sound and vibration of the struck singing bowl. Another limitation includes the lack of actigraph assessment for the evaluation of the mean reported sleep duration on work- days.

## 5. Conclusion

Despite the discrepant effect in subjective and objective outcome measures, our results suggest that the singing bowl treatment or application may be useful, especially in women, to improve subjective sleepiness. Further studies are needed to confirm this preliminary data, and to evaluate if singing bowl application might be useful in situations of intellectual focus requiring high levels of alertness.
